# GPs’ perspectives on diagnosing childhood urinary tract infections: a qualitative study

**DOI:** 10.3399/BJGP.2021.0589

**Published:** 2022-07-26

**Authors:** Hanne Ann Boon, Ann Van den Bruel, Jan Y Verbakel

**Affiliations:** EPI-Centre, Department of Public Health and Primary Care, KU Leuven, Leuven, Belgium.; EPI-Centre, Department of Public Health and Primary Care, KU Leuven, Leuven, Belgium.; EPI-Centre, Department of Public Health and Primary Care, KU Leuven, Leuven, Belgium; NIHR Community Healthcare Medtech and IVD Cooperative, Nuffield Department of Primary Care Health Sciences, University of Oxford, Oxford, UK.

**Keywords:** Belgium, child, diagnosis, general practice, parents, qualitative research, urinary tract infections, urine specimen collection, uncertainty

## Abstract

**Background:**

Diagnosis and management of childhood urinary tract infection (UTI) is challenging in general practice because of a range of factors.

**Aim:**

To explore GPs’ perspectives concerning the barriers to and facilitators for diagnosis and management of childhood UTI.

**Design and setting:**

Qualitative study in general practice in Belgium.

**Method:**

Semi-structured interviews with 23 GPs from January 2021 to June 2021 were carried out. Interviews were video-recorded and audio-recorded, transcribed verbatim, and analysed using a thematic approach.

**Results:**

The barriers to early diagnosis of UTI were the assumption of low UTI prevalence and aspecific presentation of UTI in children, difficulties in urine collection, and diagnostic uncertainty. All GPs indicated that they sampled urine in either children with specific UTI features (for example, dysuria, abdominal pain) or unexplained fever. Facilitators for UTI screening were instructional material for parents, skill training for GPs, additional nursing staff, novel non-invasive convenient collection methods, online decision support informing parents when to bring a urine sample to the consultation, and an accurate, easy-to-use point-of-care test for UTI. Empirical antibiotic treatment was initiated based on dipstick test results, clinical features suggestive of UTI, severity of illness, gut feeling, long duration of fever, time of the day, and parents’ ability to judge disease severity.

**Conclusion:**

The assumption of a low UTI prevalence, absence of obvious UTI features, and difficult urine sampling might cause childhood UTIs to go undetected in general practice. Diagnostic uncertainty makes appropriate treatment challenging.

## INTRODUCTION

Childhood urinary tract infection (UTI) occurs in nearly 6% of outpatient children aged under 5 years.^1^ UTIs may cause temporary discomfort for the child and acute complications such as renal scarring, hydronephrosis, kidney abscess, or sepsis. The long-term consequences in adulthood are not well understood, but potential comorbidities are impaired renal growth, hypertension, and end-stage renal disease.^2^^–^4 Acute complications can be prevented with antibiotic therapy,^2^^–^5 but diagnosis is challenging in general practice.

Children with UTI have fewer specific clinical features than adults with UTI. Features such as haematuria, cloudy urine, smelly urine, darker urine, frequency, or dysuria increase the probability of UTI, but are often absent. Only a few features, such as circumcision in boys, nappy rash, or the presence of stridor decrease the probability of UTI.^6^ The absence of signs and symptoms of UTI in children makes it difficult to rule out UTI at an early stage.

To date, there are no well-defined sampling strategies for GPs. Some international guidelines^2^^–^3 recommend sterile collection methods such as catheterisation or suprapubic aspiration; however, the feasibility of such methods for primary care is questionable.

Additionally, in outpatient settings, there are differences in the processing of urine samples, compared with inpatient settings. Samples are often obtained at home by the parents and then brought back to the practice. A pick-up service transports the samples from the practice to the laboratory. Therefore, the pre-analytical stage is longer compared with the hospital setting, and it is not yet well defined how this affects GPs’ diagnostic workup.

It remains unclear which factors are the most important obstacles for early diagnosis according to GPs, and how they believe diagnosis might be improved. Exploring setting-specific barriers to and facilitators for diagnosing UTIs may help develop tools to improve current practice. Therefore, the aim of this study was to understand the experiences, beliefs, and challenges of GPs for diagnosing and managing UTIs in children aged up to 18 years.

## METHOD

### Study design and registration

This was a qualitative study using semi-structured in-depth interviews. The study protocol and documents were approved by the Ethics Committee of UZ/KU Leuven (S64667). This study is reported following the Standards for Reporting Qualitative Research (SRQR)^7^ and the COREQ checklist. The research group consisted of two GPs and a medical doctor.

### Participants and recruitment

Eligible participants were Dutch-speaking GPs or GPs in training working at a general practice or a health centre. GPs were invited using flyers, paper letters, emails, newsletters, and social media. Participants were purposefully selected from those responding to the invitation to achieve a maximum of variation in sex, years of experience, location of the practice (rural: >15 kilometres from a hospital; city: ≤15 kilometres from a hospital), and type of practice (<3 GPs versus ≥3 GPs). Participants were made aware that the study was conducted to understand the challenges in diagnosing paediatric UTIs in general practice, and that the interviewer was a medical doctor conducting a PhD on UTI diagnosis in children. The recruitment ended after data saturation was reached, that is, when no new information emerged from the last two interviews. Data saturation was discussed among all members of the research team.

**Table:** How this fits in

Diagnosis of childhood urinary tract infection (UTI) is challenging in the outpatient setting. GPs’ perspectives for the diagnostic workup of childhood UTI are not well understood. In this study, it was found that, assuming low UTI prevalence, the aspecific presentation of UTI in children and difficulties in urine collection were barriers to diagnosis of childhood UTI. Diagnostic uncertainty makes appropriate treatment challenging. Factors that might improve the diagnostic workup were novel non-invasive collection techniques, instructional material for the parents, skill training for GPs, decision support tools, accurate and easy-to-use point-of-care tests, and guidance on urine culture interpretation.

### Interviews

One interviewer performed online semi-structured interviews with GPs using a pre-specified topic guide, which was developed and reviewed by the whole research team and pilot tested with a GP who did not participate in the study. The flexible topic guide contained open questions on urine sampling, UTI diagnosis, and UTI treatment in children, and was revised in response to emerging themes and new insights from previous answers (Supplementary Box S1). The interviewer had limited practical experience on UTI diagnosis and urine sampling as part of her medical training. She did not establish a relationship with the participants before study commencement; however, she already knew some of the participants because of previous participation in another study.

The interviews were conducted online using a Microsoft Teams one-on-one meeting, which was video-recorded and audio-recorded. Informed consent was registered for each participant using Qualtrics (Version 08/2021, Provo, UT, US). Field notes were made during and after every interview. Because the interviews took place during the SARS-CoV-2 pandemic, GPs were asked to respond to the questions based on usual care, that is, referring to normal clinical practice preceding the pandemic. At the end of each interview, GPs were asked if they thought the pandemic might have affected UTI diagnosis in children.

### Data collection

The interviews and data analysis were conducted in parallel, to monitor when data saturation was obtained and to allow revision of the topic guide. One person transcribed the interviews verbatim in Microsoft Word, anonymised them, and afterwards checked for inconsistencies against the original recording. If necessary, remarks on the GPs’ non-verbal communication were added to the transcript. Transcripts were not returned to participants for comments. There were no repeat interviews.

### Analyses

The analyses were performed based on the concepts of grounded theory, using a thematic approach.^8^^,^^9^ Each interview was coded into schemes using the QUAGOL-Method (Qualitative Analysis Guide developed at KU Leuven) and NVivo version 12 software.^10^^,^^11^ Each single excerpt of data was coded with multiple codes. Discrepancies in coding or interpretation were resolved through discussion among the research team. The content of the answers was coded into a common code list, which was combined by deductive coding to develop coding schemes. Next, coding schemes were combined as much as possible into main themes. The main themes were shared and discussed among the research team.

## Results

### Participant characteristics

From January 2021 to June 2021, 23 GPs participated in the study (Table 1). Five GPs declined to participate before study initiation, owing to time restrictions. There was no dropout of participants after study initiation. The GPs were aged between 25 and 66 years and came from 21 general practices located across Flanders in a rural (*n* = 10) or city (*n* = 11) area. About half of the practices (*n* = 12) consisted of GPs only while nine other practices provided multidisciplinary care. The median duration of the interviews was 38 minutes (range: 25–67 minutes).

**Table 1.** Participant characteristics

**Variables**	***N*= 23**
**GP age**	
20–30 years	7
>30–40 years	11
>40–50 years	1
>50–60 years	2
>60 years	2

**Years of medical experience**	
<5 years	6
>5–10 years	8
>10–20 years	4
>20–30 years	2
>30 years	3

**Sex**	
Female	15
Male	8

**Practice location (*n*= 21)**	
Rural	10
City	11

**Type of practice (*n*= 21)**	
Solo or duo	7
≥3 GPs	14

Most GPs thought the SARS-Cov-2 pandemic did not affect UTI diagnosis in children at their practice. Four main themes and eleven subthemes were identified. Each quote is labelled with a unique identifier for the GP.

### Theme 1: UTI prevalence assumed to be low

The majority of GPs felt that diagnosing UTI is important, but thought that the probability of UTI causing an acute illness was low in general practice:

*‘The probability that a child with fever who goes to the paediatrician’s office has a urinary tract infection is a lot higher than in our practice … simply because they see the special cases.’*

(GP2, female, 10 years’ experience, city)

*‘A urinary tract infection is for me, uhm … especially something that I always keep in the back of my mind because it is less common than an upper respiratory tract infection.’*

(GP14, male, 2 years’ experience, city)

*‘It is not so frequent that fever in young children leads to a UTI …’*

(GP19, male, 35 years’ experience, city)

### Theme 2: Urine collection challenges

#### Subtheme 2a: Adhesive bags are preferred option

In general, GPs preferred using adhesive bags over clean-catch, catheterisation, or suprapubic aspiration in infants, because the former method is non-invasive and ‘easy-to-use’. Many GPs believed that adhesive bags are more successful than clean-catch in young children. Parents can perform this method themselves at home and try several times:

*‘If it fails, we can try again … the child will not suffer much, so the threshold is very low.’*

(GP20, female, 25 years’ experience, rural)

*‘I think that is by far the easiest thing to do.’*

(GP12, male, 33 years’ experience, city)

#### Subtheme 2b: Problems in using adhesive bags

Although adhesive bags were often preferred in infants, GPs reported several disadvantages, such as high contamination rate, and the time and effort required for parents to obtain a sample owing to unsuccessful catches (leaking of the bag):

*‘It’s difficult, urine leaks out of the bag, the bag comes off …* [sighs] *and only a few drops of urine remain inside the bag.’*

(GP10, female, 5 years’ experience, city)

*‘… you have to have good-quality bags, but … you don’t have much choice.’*

(GP11, female, 4.5 years’ experience, rural)

#### Subtheme 2c: Importance of cooperation by parents

GPs indicated that urine is often obtained at home, and they felt that cooperation from the parents is important for obtaining a reliable urine sample. Some GPs were worried that parents have to put in a lot of effort to obtain urine:

*‘… it’s not difficult for me, it’s difficult for the parents.’*

(GP15, female, 5.5 years’ experience, rural)

*‘… when I propose sticking a bag they* [parents] *say, ‘’Oh no, last time that also failed.”’*

(GP18, female, 10.5 years’ experience, city)

*‘… the parents must be willing to cooperate of course, everything depends on that.’*

(GP19, male, 35 years’ experience, city)

*‘You don’t always see a parent who is motivated to come back or drive to the lab …’*

(GP6, female, 4 years’ experience, rural)

#### Subtheme 2d: Urine catheterisation unacceptable in primary care

Urine catheterisation being performed in primary care did not seem feasible or acceptable, because GPs were worried about the traumatic consequences (physical and psychological) for the child and/or parents, time required for catheterisation, and lack of experience:

*‘As an adult that’s already embarrassing. But as a child who doesn’t understand what’s happening … they have to force it. They panic … No, it is painful and then it does not work.’*

(GP10, female, 5 years’ experience, city)

GPs preferred to refer children for catheterisation to secondary care, only if they are severely ill. Some GPs were worried that catheterisation is performed too often in secondary care:

*‘I would never want to do it in my life … I think that’s way too traumatising! I don’t think a lot of family doctors are up for that … I wonder what the benefit is compared to the traumatising effects* […] *One of my own children had pyelonephritis* […] *In the hospital urine was obtained through catheterisation in a rather brutal way* […] *Even if we don’t have that impression, it can be traumatising for children in the long term … there should be an evidence-based flow to collect urine without being immediately very invasive, even in hospitals.’*

(GP7, female, 23 years’ experience, city)

#### Subtheme 2e: Clean-catch method in infants seems unsuccessful

Half of GPs were not familiar with performing clean-catch in infants, for example, direct first-stream catch of a urine sample in a urine container:

*‘I have never heard of that.’*

(GP11, female, 4.5 years’ experience, rural)

Most GPs who were familiar with this method had not tried it in practice, because they were concerned that clean-catch with or without stimulation techniques might often be unsuccessful, time consuming, complex, and messy for the parents:

*‘You can never take ten or five minutes in the hope that the child will urinate during your consultation.’*

(GP14, male, 2.5 years’ experience, city)

*‘… a little baby … constantly pushing your hand away … so I know that there are stimulation methods, um … whether they are particularly successful … I don’t think so, but that’s my personal opinion.’*

(GP12, male, 33 years’ experience, city)

A minority of GPs had tried the Quick-Wee method,^12^ or other clean-catch techniques with stimulation and found it impractical:

*‘It just doesn’t work* […] *For young children, it sometimes works … only in very young children, below nine months old.’*

(GP18, female, 10.5 years’ experience, city)

*‘I have never succeeded in doing that … it is not practically feasible, here in the practice at least.’*

(GP13, female, 6 years’ experience, city)

Some GPs would be prepared to ask parents to perform the clean-catch method at home:

*‘Maybe the parents can do it at home … with extra stimulation that’s something we can try* […] *Sometimes it doesn’t have to be difficult and simple tricks are also useful.’*

(GP22, male, 8 years’ experience, rural)

#### Subtheme 2f: Improving collection methods might facilitate urine collection

Facilitators for urine collection were an instruction sheet for parents, skill training for GPs, nurses at the practice, adaptation of available collection methods, novel non-invasive collection methods, sticking the bag themselves at the practice, or an algorithm that informs parents whether they should bring a urine sample to the initial consultation. GPs also highlighted the importance of a well-located, spacious, and attractive toilet room:

*‘I have been working for about eight years now, but … I have never had a course on how to obtain a urine sample* […] *What are the tricks?* […] *That would be very useful.’*

(GP22, male, 8 years’ experience, rural)

### Theme 3: Diagnostic uncertainty

#### Subtheme 3a: UTI features and unexplained fever drive urine analysis

All GPs claimed to obtain urine samples in two clinical situations: children with clinical features of UTI such as dysuria, abdominal pain, incontinence, and vaginal itching, or children with fever without source. Fever without source was often defined as a child with high fever, who looked severely ill, and where the clinical examination was reassuring.

Long duration of fever raised suspicion of UTI. Most GPs used a ‘wait-and-see’ strategy and advised the parent to collect a urine sample if the child remained ill after 2–3 days or longer. A minority of GPs requested a urine sample more rapidly on Day 1 when there was no focus for the infection.

Some GPs mentioned that in using this strategy they only detected severe UTI cases, because young children usually present with vague symptoms:

*‘I think we only see it in small children if they have fever, so if it’s a trivial infection, we’re just not going to see it … so I think the moment they come in with symptoms and fever, it’s by definition necessary to detect it quickly …’*

(GP11, female, 4.5 years’ experience, rural)

*‘*[sighs] *… a bladder infection occurs usually in an older child … in young children, I don’t think we diagnose bladder infections unless they have pyelonephritis* […] *We don’t take a urine sample from a child without fever under two to three years of age because they don’t complain of pain while urinating.’*

(GP2, female, 10 years’ experience, city)

*‘Uhm* […] *Especially in younger children, UTI is an incidental finding when you take a urine sample.’*

(GP9, female, 0.5 years’ experience, city)

*‘But I have already experienced it: an ear infection that always comes back and then you do a urine test and suddenly it turns out to be positive … you treat the problem and the ear infection also stays away.’*

(GP19, male, 35 years’ experience, city)

#### Subtheme 3b: Unreliable urine dipstick test

GPs felt the urine dipstick test was not reliable enough because many participants had little confidence in test results owing to fast expiration of the tests and non-concordance with laboratory results:

*‘I find the dipstick test very unreliable … it expires very quickly, it discolours when exposed to light.’*

(GP14, male, 2 years’ experience, city)

*‘I found out that if you leave the jar open too long, all the red blood cell tests are positive* [sighs]. *’*

(GP17, female, 10.5 years’ experience, city)

Another limitation were the semi-quantitative results, which they thought were more difficult to interpret:

*‘It often gives me unconvincing results, light discolouration, what do you do with that? That is difficult.’*

(GP13, female, 6 years’ experience, city)

Important advantages of the dipstick test were the user-friendliness and short analysis time, which allows early initiation of empirical antibiotic treatment if the dipstick test is positive.

#### Subtheme 3c: Manual microscopy laborious

Manual urine microscopy was rarely performed at the practice because GPs thought that performing microscopy was complex and time invasive, or that they lacked experience using the microscope and had more confidence in microscopy results from the laboratory:

*‘I don’t think that can be part of today’s general practice … you need time and time is missing.’*

(GP20, female, 25 years’ experience, rural)

Some GPs used to have a microscope, but did not use it any more:

*‘That’s all so laborious* [laughs] *… I still have a counting chamber somewhere, but I don’t think I’ve used it for twenty years.’*

(GP19, male, 35 years’ experience, city)

#### Subtheme 3d: C-reactive point-of-care test less relevant for cystitis

According to participants, the C-reactive protein (CRP) point-of-care test seemed useful to rule out pyelonephritis, but less so for cystitis. Some GPs felt that the CRP point-of-care test might be only useful for respiratory infections and not for UTIs.

#### Subtheme 3e: Long turnaround time of urine culture

The majority of GPs always requested urine culture for children as reference standard and especially to adjust empirical antibiotic treatment.

Some GPs only requested urine culture whenever the dipstick was positive. The most important limitations of urine culture were the long turnaround time:

*‘In your reasoning you have to take into account: Is it possible to wait that long or not?’*

(GP15, female, 5.5 years’ experience, rural)

Another important barrier was the interpretation when there were atypical pathogens, low colony-counts, multiple pathogens, or no urine white blood cells. Many participants believed there was not sufficient practical guidance for such cases:

*‘If you find a pathogen, but no inflammation, no pyuria or haematuria … or multiple pathogens, I sometimes don’t know what to do.’*

(GP4, male, 2.5 years’ experience, rural)

GPs believed a novel test for UTI should provide fast, easy-to-interpret, and reliable results at low costs.

### Theme 4: Empirical treatment on high suspicion of UTI and referral of severe cases

GPs initiated empirical antibiotic treatment before urine culture results when they had high suspicion of UTI, based on a positive dipstick test or presence of UTI features; and also when they believed the clinical picture did not allow awaiting urine culture results, such as high fever, impression of a severe illness, and low understanding of alarming symptoms by the parents:

*‘If it* [the dipstick test] *clearly discolours, for nitrites and high WBC* [white blood cell count] *… in a child with high fever where it is difficult to wait, then yes, I will start immediately, I will not wait for the culture.’*

(GP21, female, 6 years’ experience, rural)

*‘… for children that have had UTIs in the past … or the parents don’t really understand it either. Than you will be tempted to start antibiotics that may not be necessary.’*

(GP15, female, 5.5 years’ experience, rural)

*‘That waiting time is sometimes a problem … and then you start with amoxicillin sooner than normal.’*

(GP11, female, 4.5 years’ experience, rural)

GPs referred children to secondary care based on the impression of a severe illness, such as pyelonephritis, long duration of fever, decreased intake or dehydration, gut feeling of ‘something is wrong’, young age (<3 months), and anxious parents; or for atypical presentations: recurrent UTI, atypical pathogens, urinary tract abnormalities, and history of previous pyelonephritis:

*‘Very sick children with a positive culture, we’re going to refer rather easily, eh, in the context of pyelonephritis.’*

(GP18, female, 10.5 years’ experience, city)

*‘That’s a bit of gut feeling, you can’t really put a finger on that … often high fever, that the child falls asleep on the mom’s lap … no longer drinks anything and has no more pee puddles.’*

(GP22, male, 8 years’ experience, rural)

*‘Concerning urinary tract infections, I’m mainly going to look at what the recurrent character is, or if there are very special pathogens … so then I will refer, but let’s say for 99% of the urinary tract infections I’m not going to refer.’*

(GP12, male, 33 years’ experience, city)

## Discussion

### Summary

In this study, it was found that, assuming low UTI prevalence, absence of UTI features and difficult urine collection are barriers to detection of paediatric UTIs in general practice. Diagnostic uncertainty owing to unreliable dipstick test results, long turnaround time, and unclear interpretation of urine culture results make deciding on appropriate treatment difficult.

According to GPs, the diagnostic workup might be improved by organising skill training on urine collection for GPs, providing instructional material for the parents, developing novel non-invasive and convenient collection methods, attaching bags at the practice, recruiting nurses, implementing an algorithm to inform parents whether they should bring a urine sample, an easy-to-use point-of-care test, and guidance on interpretation of urine culture results. Most GPs rely on urine collection material (urine containers and adhesive bags) provided for free by the central laboratory, and the current options are limited.

Any novel collection method for general practice should be easy to use, non-invasive, and accurate, and should be able to be carried out by parents, as there is often no time for urine collection during the consultation.

### Strengths and limitations

Several facilitators for UTI diagnosis in children were identified. Additionally, several factors that influence GPs to initiate antibiotic treatment while awaiting urine culture results were identified. Because the interviewer had limited practical experience on urine collection and UTI diagnosis, it is thought that there were no important pre-assumptions during the interviews and analysis.

Video interviews facilitated contact with GPs who may not otherwise have been able to participate owing to time constraints and the ongoing COVID-19 pandemic.

Although GPs were recruited using purposeful sampling aiming to reach a broad spectrum of GPs, the group of participants had a low median years of experience (6 years; range: 0.5 to 41 years). This might be explained by the fact that young GPs see a lot of children in Belgium and therefore may have been more interested in the topic. Interviews were restricted to those who agreed to participate, which may have excluded GPs who were less likely to perform urine sampling in children or selected for those with more interest in diagnosis and management of UTI in children. This study did not include interviews with parents on urine collection. When GPs referred to home urine collection, it was usually for their own children, and they referred to the opinions of parents attending their own practice.

### Comparison with existing literature

These findings reflect the trend of parents preferring adhesive bags over clean-catch methods, because they perceive clean-catch as being time consuming and messy.^13^ Catheterisation is often perceived as traumatic by the parents.^14^ This might help explain why GPs also prefer using adhesive bags. In a study of semi-structured interviews with parents, parents indicated that they would prefer more guidance on urine collection,^15^ and, in the current study, GPs proposed information sheets or video clips as a way to educate parents on urine sampling and storage. Parents of children who have had a UTI often feel that there was a delay in investigation by outpatient health professionals.^15^ In this study, GPs confirmed that they often apply a ‘wait-and-see’ strategy and that they feel that diagnosis is delayed owing to difficulties in urine collection.

Another qualitative study indicated that Australian GPs believe urine collection is difficult and prefer non-invasive collection methods.^16^ Although that study found that many GPs preferred clean-catch, this could not be confirmed in the present study, as most of the GPs from this sample preferred bags over clean-catch.

### Implications for research and practice

This study highlights an unmet need for better management of UTIs in children and improved guidance on use of sampling methods by GPs and parents. The assumption by GPs that UTIs are not common in children in general practice may contribute to UTIs being missed. The diagnostic workup of acutely ill children should include more regular urine investigation, especially when the clinical features are non-specific (such as a red throat or seemingly inflamed eardrums), because non-specific features for another infection do not necessarily indicate the absence of UTI.

Additionally, more precise urine collection methods or adaptations to the available urine collection methods should be established to make urine collection more efficient in the outpatient setting. Cooperation with the parents is essential, and information sheets could be useful to assist parents in obtaining and storing urine at home before transport to the GP’s practice. Studies investigating urine collection methods for the outpatient setting should include parents and GPs as population besides study nurses. GPs should be informed about all available techniques to enhance on-site urination during the first contact consultation and minimise contamination. These methods could further support and empower GPs and parents to properly manage children with urinary tract symptoms in the outpatient setting and improve early detection of potentially serious childhood UTIs.
